# Exploring Parent Carer Empowerment in Childhood Onset Disability: A Qualitative Study

**DOI:** 10.1111/hex.70808

**Published:** 2026-08-05

**Authors:** Jim Reeder, Saira Minhas, Sharon Foxwell, Mark Williams, Jane R. Smith, Sally Kendall, Christopher Morris

**Affiliations:** ^1^ PenCRU (Peninsula Childhood Disability Research Unit), Health & Community Sciences University of Exeter Medical School Exeter Devon UK; ^2^ PenCRU Family Faculty, Health & Community Sciences University of Exeter Medical School Exeter Devon UK; ^3^ Exeter Collaboration for Academic Primary Care (APEx), University of Exeter Medical School University of Exeter Exeter Devon UK; ^4^ Centre for Health Services Studies University of Kent Canterbury Kent UK

## Abstract

**Background:**

Supporting parent carers to have greater agency and control over the decisions and actions regarding the care of their child is a central tenet of contemporary health and social care policy. This study explored contextual factors influencing the empowerment of parent carers of disabled children from the perspective of a range of stakeholders.

**Methods:**

We used focus groups and semi‐structured interviews to collect data from 28 participants: fifteen parent carers, nine children's service providers, three service managers and one commissioner. Interviews and focus groups were recorded and transcribed verbatim. Data were analysed using a process of reflexive thematic analysis.

**Findings:**

We propose a novel model that conceptualises the complex system of interrelated contextual factors influencing parent carer empowerment. This model offers a framework to present the three main themes we generated from the data: (i) Lived Experience: Roles, identities and interactions, (ii)Tensions in a Complex System, and (iii) Information, Knowledge and Wisdom.

**Conclusions:**

The complex system influences both parent carers and service providers, constructing and shaping how these roles and identities are experienced and performed. This is particularly apparent in the interactions between parent carers and service providers, where information is shared, knowledge is created and privileged, and where parent carers can be empowered or disempowered.

**Patient/Public Contribution:**

Our project team includes a group of parent carer research partners. They have been fully involved in planning and design, data generation, data analysis and co‐authorship of the paper. We have recorded involvement using the GRIPP2‐short form.

## 1 Background

Supporting service users to have greater agency over decisions relating to their own care has long been the aim of policy‐makers and service providers, both in the UK [1, 2], and internationally [3, 4, 5, 6]. Policies have sought to place individuals and their carers at the centre of healthcare decision‐making, recognising that active engagement can improve outcomes, satisfaction, and service delivery [7, 8]. Central to this agenda is the concept of empowerment, defined by the World Health Organization (WHO) as a process through which people gain greater control over decisions and actions affecting their health [9].

Empowerment is not only about gaining more agency over individual care decisions, it extends to shaping broader aspects of service provision. This might include service users being involved as active contributors in the co‐design and evaluation of services, in the delivery of quality improvement projects, or by working as research partners alongside academics and clinicians. Such involvement and engagement is underpinned by the philosophy that people with lived experience of particular health conditions are best placed to advise on what support and services are likely to have the most meaningful impact on their lives [10, 11].

Service user empowerment reflects a paradigm shift away from professional‐led models of healthcare and towards more participatory, rights‐based approaches. Yet, while these ideals are well established in legislation and policy discourse, the practical implementation of service user empowerment remains variable, particularly in complex care contexts [12, 13, 14].

An example of a complex care context is that of childhood‐onset disability. Disabled children and young people (CYP) account for around 11% of CYP in England [15] and often require coordinated, ongoing support from multiple health and social care providers. The decisions and choices made about their health and treatment can frequently have implications that extend beyond healthcare; affecting education, social care, family dynamics, and future opportunities [16, 17, 18]. Parent carers play a crucial role in mediating between their child and service providers. Under UK legislation, a parent carer is defined as any individual aged 18 or over who provides, or intends to provide, care for a disabled child for whom they have parental responsibility [19]. As advocates, coordinators, and providers of practical and emotional support for their children, parent carers occupy a unique position within healthcare systems.

Various initiatives and programmes have been developed to support the empowerment of parent carers. These include educational workshops, peer‐support networks, digital tools, and participatory forums intended to enhance parent carers' knowledge, confidence, and agency in health‐related decision‐making [20]. Despite these efforts, many parent carers continue to report feeling marginalised, overwhelmed, and disempowered when navigating healthcare systems [12, 21, 22].

This paper describes a study that is part of a programme of research that aims to understand why parent carer empowerment remains so difficult to achieve in practice, despite the favourable policy environment and the proliferation of supportive interventions. The overarching goal is to identify, co‐create and evaluate potential new ways for service providers and parent carers to work together, that might promote meaningful parent carer empowerment and, thereby improve outcomes for disabled children and their families.

A central premise of this work is that parent carer empowerment processes are deeply embedded in the broader systems and contexts in which care takes place. Accordingly, any effort to design or adapt interventions aimed at enhancing parent carer empowerment must be grounded in a detailed understanding of these contexts. This aligns with the Medical Research Council's updated guidance for developing and evaluating complex interventions [23] and with realist evaluation approaches [24] both of which emphasise the need to understand how mechanisms of change are triggered within specific contexts.

This qualitative study aims to elucidate contextual factors that influence parent carer empowerment from the different perspectives of service providers, managers, commissioners, and parent carers within a large NHS organisation in the UK.

## 2 Methods

We conducted our research with a strong commitment to methodological integrity and quality following relevant guidance and recommendations [25, 26]. We demonstrate this by highlighting the coherence between our research aim, theoretical assumptions, and the study design and procedures. Additionally, we have embedded key values of reflexivity, openness, and transparency throughout [26].

### 2.1 Patient and Public Involvement and Engagement (PPIE)

We are delivering our programme of research in collaboration with a group of five parent carer research partners, three of whom are co‐authors of this paper. The group's involvement has materially improved the delivery of the study, the detail of which is described in Table 1.

**Table 1:** Guidance for Reporting Involvement of Patients and the Public – Short Form (GRIPP2‐SF).

Topic	Item
1. Aim (Report the aim of PPI in the study)	The group of parent carer research partners were encouraged to bring their own lived experience and expertise, with the aim to: Plan and deliver the study in a way that optimised recruitment of participants from a diverse range of backgrounds. Bring different perspectives to the data generation process. Explore alternative avenues, gain additional insights and offer potential different interpretations in the analysis. Identify ideas for effective dissemination.
2. Methods (Provide a clear description of the methods used for PPI in the study)	A group of five parent carer partners was convened to support all stages of the planning, delivery and dissemination of the study. The group is from a range of ethnic backgrounds, living in UK. There are four mothers and one father. Their involvement as partners has been supported by the Family Involvement Co‐ordinator (also a parent carer) linked to PenCRU's Family Faculty at the University of Exeter. The group met regularly via Zoom to discuss the progress of the study and to share their contributions. Meeting frequency varied depending on the stage of the project. Early in the partnership, we used the Involvement Matrix as a tool to discuss and negotiate levels of involvement for this study, in the context of the larger programme of work. This allowed flexibility for each parent carer partner to choose a level of involvement that worked for them. Parent carer partners were fully involved in planning delivery strategies, developing a working definition of parent carer empowerment, designing the interview schedule/focus group plan, conducting interviews, facilitating focus groups, reviewing transcripts and disseminating findings. Provision was made to ensure the group had adequate access to necessary IT and training. For the members of the group who conducted interviews and/or facilitated focus groups, specialist training was provided. The group were also compensated for their time and any costs incurred in line recommended policy/practice.
3. Study Results (Outcomes—Report the results of PPI in the study, including both positive and negative outcomes).	The PPI strengthened the study in a number of ways: 1. **Planning recruitment and delivery strategies.** The group advised on appropriate recruitment strategies. They also suggested that provision was made for choice of involvement – focus group or interview, face‐to‐face or online. 2. **Designing interview schedule/focus group plan.** The group co‐produced the research materials, including the interview schedule and the focus group plan. This involved preparing a definition of parent carer empowerment that could be shared with participants. The group also helped to trial online software for use during the focus group. 3. **Data generation activities.** Two parent carer partners conducted interviews and supported the facilitation of the focus group. This provided variation in interviewer/interviewee dynamics, which we believe added to the richness of the data generated. 4. **Data analysis and interpretation.** Three parent carer partners were actively involved in the analysis and interpretation of the data. This involved reading and checking transcripts, offering interpretations of what they thought was going on in the data, and regular check ins with the lead researcher. 5. **Writing for publication.**Three parent carer partners have also made substantive contributions to writing the manuscript for publication in an academic journal. We believe this has resulted in an accessible paper, which is likely to resonate with a broad readership. There were also some challenges to parent carer involvement: **Additional time/resources:** The level of involvement undertaken with parent carer partners required additional time and resources, which meant that the study took longer to complete.
4. Discussions and Conclusions (Outcomes—Comment on the extent to which PPI influenced the study overall. Describe positive and negative effects)	Given the topic of our research, we felt it was vital for parent carer research partners to have authentic agency, influence and control over the decisions and actions related to this study. As such, we committed substantial planning, time and resources to ensure our PPI was both robust and meaningful. We believe the PPI in this study was effective and materially influenced important aspects of the study, based on the impacts described above. This might have been related to several factors. First, our parent carer partners received training, both associated with this project and with experience from previous studies they had been involved with linked to their membership of the Family Faculty. In addition, the support of a dedicated Family Involvement Co‐ordinator, helped to quickly establish a trusting and respectful relationship between the parent carer partners and the rest of the research team. The correct processes were in place, beginning the partnership from the planning phase of the study and ensuring there was sufficient budget for appropriate compensation. There was also a commitment from all members of the research team to a collaborative partnership with parent carers. Aside from the additional time and resources used, we do not believe our PPI produced any negative effects or influences on the study.
5. Reflections/critical perspective (Comment critically on the study, reflecting on the things that went well and those that did not, so others can learn from this experience)	We feel PPI was embedded fully in this research and believe this is a key strength of our study. We found it very helpful to use the Involvement Matrix as a tool to discuss and negotiate levels of involvement for this study, in the context of the larger project. Involving the parent carer partners throughout this early stage of the project helped to foster trusting relationships, which continue to bear fruit in ongoing work. To conduct research within the NHS, researchers need to be issued with a research passport. Typically, this is linked to a substantive contract, either with an NHS organisation or with a higher education institution. Our parent carer partners had neither of these contracts in place, which created a potential barrier to their full involvement. We managed to resolve this by creating a Memorandum of Understanding between the hosting NHS organisation and each parent carer partner. A possible limitation was that all meetings have been held on Zoom, meaning that parent carer partners have never met in‐person. Meeting face to face may have facilitated relationship building and potentially more diverse involvement. However, this form of involvement is very convenient for parent carers, and allows for involvement from more geographically diverse areas.

### 2.2 Research Design, Theoretical Framework and Epistemological Assumptions

For this study we adopted a descriptive qualitative design underpinned by a critical constructivist theoretical framework. This framework reflects our epistemological beliefs that meaning and knowledge are actively co‐constructed through interactions between individuals, each with their own unique lived experience (constructivism) [27]. We also acknowledge that such meaning‐making is shaped by broader social, cultural and institutional power structures (critical theory) [28]. Importantly this position rejects any notion of researcher objectivity, rather it embraces the *active interpretative* role that a researcher plays in both data collection (data generation) and data analysis.

This position has guided our decision to use a reflexive thematic analysis (RTA) approach to analyse our data. Established and developed by Braun and Clarke, RTA offered a highly flexible approach to identify and generate patterns of meaning (themes) across our dataset [26]. Furthermore RTA provided a methodologically robust framework to approach our critical interactions with the data. This is described in detail below.

### 2.3 Researcher Positionality and Reflexivity

Given our epistemological assumptions, specifically regarding our active influence in data *generation* and meaning‐making processes, it is important that we are transparent about our positionality as members of the research team [29]. This includes acknowledging our diverse roles, backgrounds, experiences and values, in order to critically reflect on how these have influenced different aspects of the research process. To support this transparency, we have shared a brief description of each member of the research team (Table 2).

**Table 2:** Researcher Positionality.

JR	I am the lead researcher, currently undertaking a PhD, and have published previous work on partnership working with families. I have past experience of conducting semi‐structured interviews for research and I completed additional training for facilitating online focus groups. I have a clinical background, having worked as a children's physiotherapist for more than 20 years. I am also a father of three typically developing children.
SM	I'm a parent carer with lived experience navigating the Special Educational Needs and Disabilities (SEND) labyrinth. As part of the research team, I underwent specialist training in conducting interviews and in facilitating online focus groups. I carried out interviews with both professionals and fellow parent carers, and contributed to the focus groups. I brought a family perspective and real‐world insight to the development and interpretation of the study.
MW	I am the main parent‐carer to three children, two of whom have additional needs. I have had many years of interacting with statutory health services, and countless healthcare professionals representing numerous clinical specialisms, at primary, secondary, and tertiary levels. In addition to my caring responsibilities, I am a counsellor working in private practice and the third sector.
SF	I am a parent carer to a child with complex epilepsy and a severe learning disability. I have been navigating medical, social care and education systems since my daughter was diagnosed 10 years ago.
JS	I am an experienced health services researcher with a background in psychology/behavioural science and mixed‐methods development and evaluation of complex interventions. I had a son with a genetic neuromuscular condition who died as a baby and gained some insight into healthcare and third sector services supporting the care of children with complex needs during his life and through friends. I am also mother to two healthy children.
SK	I am an experienced health care researcher with a special interest in children, families and parenting. I am a health visitor by background and have worked professionally with families where a child has a complex disability.
CM	I am an experienced health services researcher and former allied health professional. I have a nephew with profound learning disability who is now in aged his thirties.

In addition, throughout the research process, the lead researcher (JR) kept a reflexive journal to document his thoughts, analytic decisions, emotional responses and emerging interpretations. This also included recording the impact of any discussions with the wider team. This practice enabled ongoing critical self‐reflection and helped to ensure transparency and methodological rigour, in line with the RTA approach.

### 2.4 Setting and Sample

We recruited 28 participants from a population of interested parties associated with the Children and Families Service in a large community based NHS organisation in the UK. There was a mix of parent carers (*n* = 15), service providers (*n* = 9), service managers (*n* = 3) and one service commissioners (Table 3). We sought representation from all relevant groups and ensured that the opportunity to participate in the study was open to all, whatever their sex, gender, race, religion, language or socioeconomic background. We achieved this using a combination of convenience sampling, volunteer response sampling and purposive sampling.

**Table 3:** Participant characteristics.

#	Group	Age	Gender	Ethnicity	First language	Child's diagnosis	Job Title	Choice for participation	Interviewer
P1	Parent Carer	35–44	Female	White British	English	Cerebral palsy, autistic spectrum disorder (ASD).		In person Interview	JR
P2	Parent Carer	24–34	Female	Asian (other)	Nepalese	ASD		Virtual interview	JR
P3	Parent Carer	35–44	Female	White British	English	Cerebral palsy		Virtual interview	JR
P4	Parent Carer	24–35	Female	White British	English	ASD		Virtual interview	JR
P5	Parent Carer	35–44	Male	White and Black Caribbean	English	ASD		Virtual interview	JR
P7	Parent Carer	35–44	Female	White British	English	Spinal muscular atrophy type1		Virtual interview	JR
P8	Parent Carer	35–44	Female	White British	English	ASD		In person interview	JR
P9	Parent Carer	55–64	Female	White British	English	Cerebral palsy		Virtual interview	SM
P10	Parent Carer Service Manager	45–54	Female	White British	English	Complex seizure disorder	Speech and language therapist	Virtual interview	JR
P11	Parent Carer	35–44	Female	White other (USA)	English	ASD		Virtual interview	SM
P12	Parent Carer	24–34	Female	White British	English	Complex undiagnosed condition		Virtual interview	JR
P13	Parent Carer	45–54	Female	White British	English	ADHD/Autism		Virtual interview	JR
P14	Parent Carer	35–44	Male	Indian	English	Chromosomal abnormality		Virtual interview	JR
P15	Parent Carer	35–44	Female	Indian	English	Chromosomal abnormality		Virtual interview	JR
P16	Parent Carer	35–44	Female	Other Black British	English	Neuro‐fibromatosis		Virtual interview	JR
SP1	Service Provider	35–44	Female	White British	English		Occupational therapist	Virtual focus group	JR/SM
SP2	Service Provider	45–54	Female	White British	English		Occupational therapist	Virtual interview	SM
SP3	Service Provider	24–34	Female	White British	French/English		Speech and language therapist	Virtual focus group	JR/SM
SM4	Service Manager	55–64	Female	White British	English		Speech and language therapist	Virtual interview	JR
SP5	Service Provider	35–44	Female	White British	English		Speech and language therapist	Virtual focus group	JR/SM
SP6	Service Provider	24–34	Female	White British	English		Therapy assistant	Virtual focus group	JR/SM
SP7	Service Provider	35–44	Female	White British	English		Occupational therapist	Virtual focus group	JR/SM
SM8	Service Manager	45–54	Female	Indian	Malayalam		Physiotherapist	Virtual interview	JR
SC9	Service Commissioner	45–54	Female	White British	English		Deputy Director of children's services	Virtual interview	JR
SP10	Service Provider	45–54	Female	White British	English		Clinical nurse specialist	Virtual interview	JR
SM11	Service Manager	55–64	Female	White British	English		Speech and language therapist	Virtual interview	JR
SP12	Service Provider	55–64	Female	Indian	Urdu		Consultant paediatrician	Virtual interview	JR
SP13	Service Provider	35–44	Female	White British	English		Consultant paediatrician	Virtual interview	JR

For the parent carer group, our recruitment strategy involved clinicians and clinical support staff in the Children and Families Service identifying potentially eligible participants as they brought their child or young person to clinical appointments. These staff received training on the study eligibility criteria (Supporting Information: Material 1), including guidance on which diagnoses and health conditions were classified as neurodisability. Staff used these criteria to determine suitability for the study, before sharing an easy‐read participant information sheet and asking the parent carer to sign a consent to contact form. Any parent carer who provided this consent was then contacted by a member of the research team (JR) who shared more detailed information, and our reasons for doing this research. If the parent carer decided to participate, their informed consent was documented. We also recruited participants through distribution of posters and leaflets, and through word of mouth.

We recruited other participant groups (service providers, managers and commissioners), via targeted emailing, presentation at staff training and through distribution of posters and leaflets.

Participants were offered a choice of whether to take part in‐person or online, and whether to take part in a focus group or interview (Table 3).

### 2.5 Data Collection

In this study, data were generated through both focus groups and individual interviews. We chose focus groups for two reasons. First, this method generates data from *interaction* and discussion between participants, which is thought to uncover aspects of the phenomena of interest that may otherwise be more difficult to access [30, 31]. Second, focus groups offer a pragmatic and efficient means to gain the thoughts and perspectives of a greater number of participants [31].

The rationale for also offering individual interviews was primarily to ensure we could accommodate potential participants who were either unwilling or unable to join a focus group [30]. However, interviews also provided an opportunity to iteratively explore nuanced aspects of the phenomena in more depth.

In order to facilitate interaction between participants with both similar and differing perspectives, our initial plan was to run three focus groups: one for parent carers, one for service providers, managers and commissioners, and a third mixed group [31]. However, prioritising participant choice and accessibility took precedence over achieving these planned group compositions.

We acknowledge that focus groups and interviews can generate different types of data. However, given our epistemological position that all data are co‐constructed through interaction and our commitment to a reflexive analytical approach, we contend that combining these data sets is both methodologically robust and likely to enhance data richness and analytical depth [30]. This position is explicitly supported by proponents of RTA, who emphasise the flexibility of the approach and its suitability for integrating data generated through a very wide variety of qualitative methods [26].

We collected data from one focus group (*n* = 5) and 23 semi‐structured interviews. The focus group was conducted online, using video conferencing software. It lasted 90 min and was facilitated by a researcher and a parent carer partner (SM and JR). The interviews were conducted either online (*n* = 21) or in person (*n* = 2) by one member of the research team (either SM or JR) and lasted between 40 and 75 min. At the beginning of the focus group and each interview, we introduced ourselves, offering a short account of our role/s and motivation for doing the research. We used a provisional interview schedule and a focus group plan (Supporting Information: Material 2), which were developed and tested during meetings with parent carer partners, to guide the data collection processes. This included a modification of the WHO definition of *patient* empowerment [9] as a conceptual description of *parent carer* empowerment.

All interviews and focus groups were recorded and audio recordings were transcribed verbatim. In the focus group, a virtual discussion board was used by participants to record additional thoughts and ideas. Transcriptions and discussion board posts were redacted to remove any identifiable information, before being uploaded to NVivo Software for analysis.

Given our epistemological beliefs, which are embedded in the RTA approach, decisions about sample size were taken in‐situ, informed by an interpretive and pragmatic judgement, rather than by a potentially problematic, neo‐positivist notion of ‘data saturation’ [32]. Guided by the diversity of our participant demographics and the time constraints of the project, we stopped further data collection after 28 participants had taken part. At this point we felt we had collected sufficient quality data to generate/construct a coherent, compelling and useful answer to our research question.

### 2.6 Data Analysis

Data analysis was led by the lead researcher (JR) who took responsibility for each stage of the analytic process. This was supported through regular discussion with the rest of the research team, not to reach consensus about analytic activities (e.g. coding or generating themes), but to gain alternative insights and potential for different understandings [26]. JR's reflexive journal documented all analytic decisions, providing an auditable trail of the analytic process. We present relevant excerpts from the reflexive journal in the results section below.

Each stage of the analytic process is summarised in Table 4, including a description of how the broader research team was involved and any points of reflexivity that were considered.

**Table 4:** Reflexive thematic analysis process.

Stage of RTA	Actions of lead researcher	Involvement of broader research team	Points of reflexivity
Familiarisation	Reading and re‐reading the transcripts. Becoming close and familiar with the dataset. Critical engagement with the dataset (this included exploring the impact of the different interviewer/interviewee dynamics). Producing a summary of the whole dataset, a ‘meta‐comment’.	Several members of the research team (SM, MW, SF) read through a selection of transcripts and JR's meta‐comment. They then provided their thoughts and opinions on ‘what was going on’ in the data.	Why might I be reacting in this way? What ideas does my interpretation rely on? What different ways could I make sense of the data.
Coding	Data were systematically coded line by throughout the dataset. The coding process was inductive. Initial codes were at a semantic level (explicit/descriptive/staying close to the data). As coding continued, codes became more latent (implicit/interpretative/moving beyond the data).	As codes became more latent, JR shared his ideas and interpretations with the research team. The purpose was to determine if the latent codes held up to interrogation and to explore if the codes might be interpreted differently by others.	What theory/ies underpin the more latent codes? Would others interpret the semantic codes differently to me? And why might that be?
Generating themes	Codes were organised into clusters with a shared central concept/idea. Early (messy) thematic maps and candidate themes were generated, which offered a possible interpretation of what was happening in the data.	JR shared early conceptual ideas/maps and code clusters with the whole research team. The rest of the team offered feedback; checking candidate themes for coherence, challenging interpretations and making suggestions for possible alternative clusterings and/or revisions to conceptual maps.	What has informed the clustering/grouping of codes? What theory (if any) inform the meaning? Is this what I was expecting? If so, how can I demonstrate that meaning making was not predetermined? If not, why?
Reviewing, developing and naming themes	Candidate themes were checked back against all the codes to ensure viability (i.e. that there was enough meaningful data to evidence the theme). Themes were then checked back against the full dataset for fit and to see if they still made sense in the original context. This also helped to identify any exceptions or possible alternative interpretations.	JR shared revised, substantive themes with the research team. We worked together to identify a clear name and succinct description for each theme. We also discussed how the themes worked together and fit in the overall analysis.	Am I trying to massage the data into a pattern that really isn't there? If so why? What does this say about my intentions? What am I missing or not noticing here?
Writing the report	The final themes and conceptual map were drawn up and a first draft of the report was written.	JR shared the draft of the report with the research team who offered feedback and corrections.	What is the report saying that is useful? And who is it useful for? How can I check if the report shares the new knowledge in a clear and easily understandable way?

### 2.7 Ethical Considerations

Cambridge South Research HRA Ethics Committee approved the study and research materials (ref‐24/EE/0020). All participants gave their informed consent to take part and were made aware of their right to withdraw at any point without consequence. Given the potential emotional sensitivity of the topic, interviews and focus groups were conducted with care and flexibility. Data were anonymised and securely stored in accordance with General Data Protection Regulation (GDPR) and institutional guidelines.

## 3 Results

We present the findings as our interpretation of the data and their meaning, using a combination of data extracts and analytic narrative. Our interpretation/analysis is elucidated with vignettes from the reflexive journal. Data extracts are labelled with a code that indicates participant group (i.e., P – Parent Carer, SP – Service Provider, SM – Service Manager, SC – Service Commissioner).

We propose a novel model that conceptualises a complex system of interrelated contextual factors that influence parent carer empowerment. We explain, justify and explore the potential utility of this model through the presentation of our three main themes: (i) Lived experience: roles, identities and interactions, (ii) tensions within the complex system, and (iii) information, knowledge and wisdom (Table 5).

**Table 5:** Summary of themes.

Theme:	Summary:
(i) Lived experience: Roles, identities and interactions.	Contextual factors in the complex system exert forces which influence expectations and standards for how roles and identities of parent carers and service providers should be performed. Thereby shaping their behaviours, actions and interactions.
(ii) Tensions within the complex system.	Forces in the complex system are sometimes in opposition, creating uncertainty confusion and tension for both parent carers and service providers.
(iii) Information, knowledge and wisdom.	There are processes by which information is accessed and shared that create and privilege knowledge, and influence how that knowledge is understood and applied.

### 3.1 The Complex System

Our initial approach to organising and interpreting our data was guided by systems thinking [33, 34]. This is grounded in the understanding that an individual's behaviours and actions do not occur in isolation, but are shaped through ongoing interactions with the broader systems and contexts in which they operate [35]. This approach provided a useful framework for interpreting participant accounts of their lived experiences, helping us to identify contextual processes, pathways and influences that may lead to either empowered or disempowered parent carer behaviours and actions. Importantly, we recognised that the behaviours and actions of both parent carer participants, and those of participants from other groups, are part of, and embedded within, a complex system of these contextual influences.

**Figure 1:**
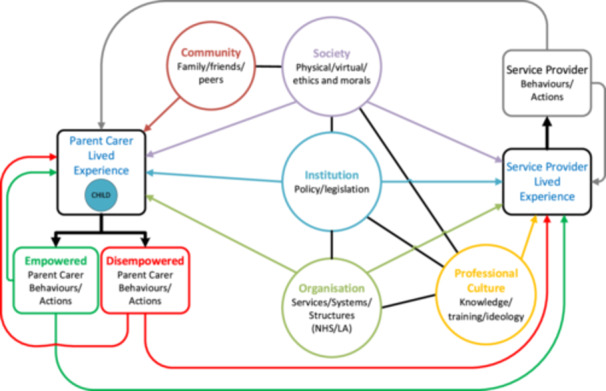
A conceptual model of a complex system of contextual factors that influence parent carer empowerment.

The conceptual model we present offers a representation of a complex system of contextual factors that influence parent carer empowerment (Figure 1). It is the result of several iterations of grouping, organising and refining of codes, supported by ongoing discussions within the research team. It is important to stress our belief that the influence/s of these contextual factors are not singular, nor readily predictable, rather they are multiple, interwoven and interdependent. It is not our intention to try to isolate discrete contextual factors and to predict or explain their impact on the parent carer experience. We do not think this is either useful or indeed possible, and attempting to do so would belie the messy, interrelatedness of the contextual factors and their potential influence on the complex system. Instead, we present our three main themes, which underpin and give real‐world context to the conceptual model.

### 3.2 Lived Experience: Roles, Identities and Interactions

Parent carer participants shared stories about their experiences, focussing on when, why and how they felt empowered. Participants from other groups, such as service providers, managers and commissioners, offered their perspectives on parent carer empowerment, identifying various factors they believed either promoted or undermined it. To make sense of these viewpoints, we found it helpful to frame our analysis around the lived experience of parent carers, exploring how this might be shaped by different contextual factors. We observed that behaviours and actions perceived as empowered or disempowered were often a product of this lived experience. In Table 6 we share data extracts which illustrate how some of the contextual factors seem to influence the parent carer lived experience.

**Table 6:** Contextual factors affecting the lived experiences of participants.

Contextual Factors	Illustrative quotes about factors affecting the parent carer lived experience	Illustrative quotes about factors affecting the service provider lived experience
Community	…sometimes it's a single parent coming into an appointment…you know they don't have a lot of support, they don't have their community…they maybe feel really isolated… – SP6 …I learned so many things I didn't know…just the parents alone were amazing…because they were like, oh, I am dealing with that, I have tried this, oh this is amazing…it was like, for once I was sitting there and somebody else was sitting with their cup of tea and we are going, this is…yay…this is amazing…” – P11 …sometimes there are parent groups, and they have a conversation amongst themselves, and then that can interfere in the professionals delivery of care. Like comparing the service they are getting, and that sort of thing… – SM8	
Society	…I think society has changed. We are no longer necessarily living near our families. We don't necessarily have in‐built support. Most parent's mums and dads both work so there's big societal shifts as well… – SP10 **…**Yeah, but she wants to get out on the rides and she's not allowed on the rides…so she feels like she's being left out. Sad, isn't it?**…**I feel like there should be…they need to include, obviously, disabled children, but it's hard. when you can't include them in everything… – P3 …it does worry me…because even though there is laws in place that will prevent him from being discriminated against…it doesn't mean it doesn't happen…it doesn't mean it is not going to happen to him… – P4 it is because of society…because of the way society at present views a learning difficulty or a mental disorder…even now…and it had come leaps and bounds since I was a kid, but it is still not socially accepted in a way… – P4	People expect ‘cures’ from health professionals and for the health professional to direct care – Focus group discussion board. …I think there's still that whole doctor/whitecoat definitely, and it stems from the whole medical model, it stems from that hospital type setting, where all of us… like I'm the same, if I go into hospital I will immediately become less confident in questioning things – SP2 … So, for example, if I go to a doctor I always consider him as a bit superior to me. He's the expert and he's going to tell me things, and I'm going to do what he says. – SM8
Institution	…you know…they haven't got the money to do certain things…which means that…you know…they either become stricter, which is what I actually believe is happening with my son and the EHCP…so I believe that if they didn't have that restriction…so like and that issue…they wouldn't be so…like have such a high bar…to try to get over… – P5 …So the only way you're gonna get afterschool care is if you've got direct payments for a PA. Oh and that's a complete nightmare…I've got loads of families who have gone through that whole thing, and then they can't find a PA. So they've got the funding in place but they can't access it… – SP13	…so the NHS definitely have a big push on personalised care, so the theory is there but, operationally, it's extraordinarily challenging… – SC9 **…**They put barriers in the way, what's the research behind it? What's the evidence? Has it been through NICE guidance? Is it MRHA, or whatever?… – SC9
Organisation	…A little bit more flexible as services, because physio for example, he's been offered several appointments, they've gone out to his home to support him. He's been asleep because of all the seizure activity, and the medication he's on, so they've discharged him… – P10 …for some people you can't even get an appointment sometimes…so for you to, for them to be saying or for this to be saying, you can do this…well you have got to get there first…you have got to actually get in contact with somebody… – P1 …that was a mine field, trying to figure out where I could put him into nursery…like…a lot of that stuff, at least from what I can tell…is not written down…I couldn't find extra…you know…extra needs…extra support…extra anything for nurseries… – P11 …They can't do anything but they can't refer us on or refer us back into the system. So we go to the single point of access. I actually emailed them in crisis. I've tried to phone, couldn't get through, emailed them in crisis and said help…We're at breaking point again. Nobody rang me back. It took three days for someone to ring me back… – P13 ……so there is very limited opportunities I feel, to be able to access services…umm…and then if you miss them, then you have to wait a long time…I mean it is not always going to be convenient for everyone at the same time… – P5	…I think the time limits on some appointments are ridiculous. I think the way the clinics – I'm just thinking of my work but having a very set time that you can only see somebody within and then it's straight onto the next person, that doesn't allow for any thinking time… – SP10 …No, I don't think it's very good. I think they're overworked, they haven't got enough time…but specialist equipment, how long it takes to arrive, how long it takes for someone to come out and adjust it, you're waiting ages… – P3 …But this is where the clinics are so rigid and, for some conditions, you can't see [the families] sooner than you want to. This is where the system fails, the interaction, rather than – I think some physicians could do better if the system was more flexible… – SP12
Professional Culture		…Some families are open, and they say that they are struggling, but I don't really know where to direct them…I think that's beyond the scope of what we should be doing. I think we should be concentrating on the child… – SM8 …they would probably still be feeling like, ‘Well actually, I'm the expert,’ and I suppose maybe intimidated by letting go of their expertise, because that's what defines you… – SP2 …But as professionals I think there's a bit of an arrogance sometimes that we're the only people who can help you… – SM4 …being open, friendly, and professionally approachable is different to a friend. I think maintaining a friendship with the patient group is not healthy… – SM8

One key contextual factor was the interaction between parent carers and service providers. The behaviours and actions of service providers appeared to have a powerful influence on parent carer lived experience and thereby their sense of empowerment (Figure 1). Whilst this was a common narrative in many accounts, it is particularly evident in P10's story.

She listened, and she showed emotion…and she got where I was coming from. She got the impact his condition was having on me and him, and our wider family, she got it, she understood it, and she empathised. She was real, she was human….

– P10

As we examined participant accounts of these interactions more closely, we began to see that service providers' behaviours and actions were a product of their own lived experience, and that this was shaped by the same complex system that influenced parent carers. This insight led us to broaden our focus; although we had initially centred our analysis on the lived experience of parent carers, it became clear that understanding the experiences of other actors in the system, particularly service providers, was equally important. An extract from the reflexive journal illustrates how this line of thinking began to take shape (Vignette 1).

Reflexive Vignette 1 1 … in our conversations with participants, the focus always seems to be on the parent carer experience. Given the apparent importance of the interaction between parent carers and service providers, I wonder if we need to begin to pay more attention to the service provider experience? How this informs the way they behave, act and interact with parent carers. It is also perhaps important to remember that service providers too are individuals, people with unique experiences, subject to being (differently) influenced by a variety contextual factors

Table 6 also presents some data extracts to illustrate how contextual factors might influence the service provider lived experience.

Building on these ideas, we returned to the data and noticed that the lived experience of both parent carers and service providers seemed to shape how they understood their roles and identities. These understandings, in turn, gave rise to expectations and standards about how they and others should behave, what actions were considered appropriate, and what fell outside the boundaries of their respective roles.

### 3.3 Tensions Within the Complex System

As we continued to explore participant accounts of their roles and identities, we noticed that multiple influences/forces act upon the lived experience of parent carers and service providers simultaneously. Some of these forces are dominant, creating firm ground and seemingly fixed roles and identities. At the same time, there are other forces which can act in tension to the dominant ones, creating role uncertainty for both parent carers and service providers.

Sometimes these oppositional forces are visible and explicit, for example in the account of SC9:

the NHS definitely have a big push on personalised care, the theory is there but, operationally, it's extraordinarily challenging … as money gets tighter and tighter across the organisations…you're having to account for every penny…and whilst we do that, individualisation and the opportunities for that get squeezed out.

– SC9

SC9 describes how difficult it has become to provide individualised care within a system that increasingly demands standardised services, where tightly defined packages of care need to be costed and paid for. We felt this highlighted a tension between very visible forces rooted in institutional policy and professional culture, and those related to organisational processes and structures. Given the explicit nature of these forces, SC9 was able to recognise the challenges associated with such conflicting demands and begin to consider how to address them.

In other participant stories, we noticed forces that were more implicit and taken‐for‐granted. A good example is evident in P14's account of his perceived role as a parent carer.

…I don't think the collaboration should go to a stage where the parents and the family are making the decisions and influencing decisions too much either…I mean what would I contribute?… Am I helping process in any way?

– P14

Despite systems and practices designed to encourage a more collaborative approach, P14 appeared firmly committed to the belief that the service provider knows best and should be the one making decisions, arguing that parents should not ‘*get in the way’* of the ‘*expert*’ decision‐making. P14's views were consistent with other parent carer participants (Table 7).

**Table 7:** Extracts from parent carer participants relating to ‘service provider as expert’.

P1	*“…you're the professional, you know what you are doing, so you are telling me what I should be doing.”*
P11	“*…maybe if somebody had just taken the time to say…you know what, I think we should look at this…instead of just, you don't know what you are talking about because you are a mum…”*
P8	*“…I don't think I had a voice because I was classed as younger mum…so it was always just don't say anything, because it will just come back to, you know…you are just a young parent…”*
P5	*“…in the appointments…I'm led by them…if I think something different I can certainly raise that but, I can't really raise much outside of what they are suggesting…because at the end of the day they are the professionals.”*

We believe the assumption, that service providers know best, stems from the persistence of traditional, professional‐led models of health care, and historic societal conditioning that privileges medical or scientific knowledge. Given the tacit nature of this assumption, it is more difficult to notice, and is perhaps why it continues to stand in tension with more contemporary, collaborative approaches to health care.

As we reflected on this, we considered the potential impact this discourse might have on the service providers (Vignette 2).

Reflexive Vignette 2 1 … when parent carers expect service providers to provide diagnoses, make decisions and offer advice, it is perhaps understandable that those service providers may feel compelled to meet those expectations. By responding in this this way, is it possible that this will reinforce their perception of their role as ‘expert’ and ‘decision maker’? I wonder what legacy this dynamic might have on the service provider's professional identity, and how this might influence future interactions with other parent carers?

We suggest that these types of parent carer behaviours can create a significant tension for service providers. On one hand they are being encouraged by institutional policy and professional standards to work collaboratively with parent carers, sharing responsibility for decision making to ‘co‐produce’ care. On the other hand they are being positioned by parent carers as the expert, expected to make decisions and, perhaps more importantly, to have all the answers.

In our analysis of participant accounts, we noticed several examples of these tensions. Often they were a consequence of a mixture of multiple forces, some visible and others hidden, but all shaping the lived experience, roles and identities of those involved. The possible implications of these tensions are explored in more detail in the discussion section below.

### 3.4 Information, Knowledge, and Wisdom

Our final theme explores how various forces in the complex system influence the movement and processing of information between parent carers and service providers. We present this theme through two sub themes. First, we explore how information moves; how it is accessed, controlled and shared. Second, we present our interpretation of how information seems to be processed and understood to become knowledge and how it is then applied as wisdom.

### 3.5 Movement of Information

Participants described how information moved in the complex system, highlighting that typically the direction of flow was from service provider to parent carer.

…she explained everything she was doing, what she was going to observe…and when she finished her observation…she was like right, I think he needs some speech therapy, I also think we need to do a referral for autism because he is showing some traits that are conducive of autism…so I was like, Oh OK…

– P4

…the first thing that comes to my head is probably [parent carers] actually being well informed and knowing…about processes, informed about therapy, informed about who they can go to …I do think empowerment is basically parents being well informed…

– SP3

These two extracts illustrate a shared expectation, between parent carers and service providers, that information should flow predominantly from service provider to parent. As we explored this further, we noticed that some service provider participants tried to limit what was available/accessible to parent carers.

…you pick and choose which information to tell them…because as [SP3] said they don't necessarily need to know all the background…that is my bag

– SP5

…I suppose there's a screening process of kind of what I think's gonna be useful and I suppose there is an element of the hierarchy to have me deciding what's useful for parents.

– SP13

Although likely unintentional, such actions could be interpreted as paternalistic, reflecting an attempt by service providers to control or gatekeep the content and flow of information to parent carers. We noticed this had a tangible impact on parent carer participants, some of whom reported feeling that information was being kept from them, leaving them out of the loop, or kept in the dark.

…I knew she was holding something back; I could see it on her face, I just knew the way she was presenting information, that there was something. But I didn't really know until a lot, lot, further down the line, that they thought he had [named syndrome].

– P10

I feel like, as a parent it's down to me to take them to their appointment, it's down to me to follow what they say… It's down to me to say this has changed, or this is new, or this has got better, but actually I feel like there is a degree of stuff that goes on behind the scenes that we don't see.

– P12

Another example of this appears in P10's and P12's stories, where both were advised not to search online for information about their child's condition. Interestingly this warning did not stop either from looking online.

she'd say to me, ‘No, don't read anything online, because it's all very old information, there's lots of new medication out there now. We don't know how his condition is going to evolve. Don't read anything.’ That's what she said to me.

– P10

“she said, ‘Don't go and Google it.’ Obviously, I went and Googled it.”

– P12

We reflected on what the impact of this might have on the relationship between parent carers and service providers (Vignette 3).

Reflexive Vignette 3 1 … I'm wondering about the implications of service providers advising parent carers not to search for information online. In reality, it seems many parent carers are likely to do this anyway. One consequence of outlawing this behaviour could be that parent carers become less willing to discuss the information they find with service providers, which might also impact the level of trust in the relationship…

These encounters offer another insight into how roles, identities and relationships are shaped in the system; potentially reinforcing once more the shared belief that the service provider is the expert and they are the only dependable source of valid information.

For the most part, participants' accounts followed the predominant narrative, that information tends to flow from service provider to parent. However, as we continued to explore this theme, we began noticing moments when there was potential for information to move in the opposite direction, from parent carer to service provider. These moments were often less explicit, hidden within parent carer stories about their interactions with service providers.

…parents feel they can't challenge a provider… if a provider says, ‘oh try this’, and a parent is like ‘well that wouldn't work for my child’…I feel like a parent wouldn't sit there and say that to a provider…they would take that away and actually go and try again with the child…knowing it probably wouldn't work…

– P4

…when she said we are going to do this and this…it instantly made me feel sick, because I thought I'm going to have to tell her that I am not happy with that…and it waited for the next phone call for me to say,'I don't think that is going to work'…– P8

In the second extract, P8 seems reluctant to share her information, perhaps underpinned by her perception that she should be “*just a parent sitting quietly”*.

### 3.6 Understanding and Applying Information

As we continued to explore how information flows through the system, our focus shifted toward how that information is actually used. Parent carer participants often spoke about having access to large amounts of information, but sometimes needing support to make sense of it.

…you can sit there and read it 5 or 6 times and still not understand what it is saying…whereas if you sit there, talking with someone…they can explain it in a simpler way that you can understand…

– P4

In P4's account, she emphasised the value of speaking with someone who could help her to interpret the information she already had, enabling her to understand and apply it effectively. This was reflected in the accounts of SP3 and SP1, who recognised that their role might need to go beyond simply providing of information.

we can give information can't we, but whether they understand that or not…it's different…and then you need to work out if you need to give the information in a different way so that they can understand it…

– SP3

…I have definitely experienced lots of people where they just want to know…tell me information…but actually its…sometimes it isn't having more information, it is about understanding the information they already have…

– SP1

As we tried to make sense of these dynamics ourselves, we reflected on the process through which information is not only received, but also is understood and applied. Viewing the data through this lens helped guide our analysis of additional participant narratives, particularly those detailing how information shared by parent carers was interpreted and applied by service providers.

Although they were often asked for information, parent carer participants felt that this had little impact on the actions of service providers or on their child's care.

**…**I had a big meeting yesterday with seven professionals and I just feel like they'd all agreed about mainstream and there's me, they're trying to sway me…I'm thinking, I just feel like I'm not getting heard enough, if you know what I mean?

– P3

Being asked to attend a meeting, P3 had the expectation that the information she shared would influence the decisions made about her child; however she was left feeling that she was not being listened to. We began to explore other participants accounts, to try to understand why the process of collecting information from parent carers may fail to translate into meaningful action.

**…**I think it's important that we're listening to families but there has to be a purpose for that doesn't it? There's no point listening if we just then merrily go on our way…You're being listened to and what you're saying is then having an influence on the practitioner…

– SM4.

In SM4's account, she reflects on the purpose of asking for information from parent carers, emphasising that it should meaningfully influence what happens next. Without this, she warns, the process risks becoming a *“tick box”* exercise. Similarly SP3 openly questions the ethics of asking parents for information that will have no real impact on her decisions or actions.

…I think that is a really sticky point for me because… when a child comes to you and the parent says [what they want to achieve]…OK great that is patient centred and I will obviously make sure that I try to do that…but realistically I am going to do the same thing I was going to do anyway, because I know and have that knowledge…so that feels wrong in my head…because I know we are trying to be really patient centred…

– SP3

In this example, it appears the information shared by the parent carer in not influencing SP3's decision‐making. The information is not being processed/understood or applied.

At times, we observed more subtle examples where information shared by parents did not appear to genuinely influence care decisions.

…but the parent comes back to you and says, ‘No, but it's happening much more frequently,’ then you say, ‘Okay, let's just do an EEG,’ you might give in

– SP12

Here SP12 indicates that she does ask for information from parent carers; however, her language suggests she is doing so less to influence her actions and more to placate the parent carer, to give the impression that she is listening.

## 4 Discussion

This study found that a complex system of interrelated contextual factors influence both parent carers and service providers, constructing and shaping how these roles and identities are experienced and performed. This is particularly apparent in the interactions between parent carers and service providers, where information is shared, knowledge is created and applied, and ultimately, where parent carers can be empowered or disempowered. In this section we position our analysis in the context of existing literature, explore possible implications for practice and identify avenues for future work in light of strengths and weaknesses of the current study.

### 4.1 The Complex System

The model we present illustrates how various social, societal, institutional, and organisational forces act simultaneously on both parent carers and service providers. Our findings reveal that these forces shape how both groups construct their roles and identities, which in turn influence their behaviours, actions, and importantly their interactions.

The model is grounded in our interpretation of the data; however, it has theoretical underpinnings in systems theory and behavioural science, particularly Bronfenbrenner's Social‐Ecological Theory [36] and Family Systems Theory [37]. As such, our model does share some characteristics with existing frameworks in child and family healthcare [38, 39, 40]; however, it offers a distinct perspective through its focus on parent carer empowerment. Unlike many traditional models that place the child or family at the centre, our model deliberately shifts the focus to the dynamic interaction between parent carers and service providers, highlighting its critical role in influencing empowerment. Here the model shares some of the ideas set out in Ralph Stacey's concept of ‘Complex Responsive Processes of Relating’ which moves beyond complex systems thinking to highlight the critical role of human interaction in organisational change [41].

Our model and findings align well with existing literature on both professional and personal identity, reinforcing the idea that identity construction is shaped by multiple, complex, and interrelated factors [42, 43]. These influences can sometimes conflict with one another, leading to role tension and ‘identity dissonance’ [44], particularly among service providers. We propose that when service providers experience this ‘shaky professional identity’ [44], they may revert to the familiar territory of the traditional medical model, where they are positioned as the expert and can retain agency and control over decision‐making. These behaviours and actions, in turn, shape the parent carer experience and are likely to contribute to their feeling of disempowerment.

This example highlights an important finding of our study; that the construction of parent carer and service provider roles and identities appear intertwined and contingent on their interaction. We argue that this interaction is performed within, and shaped by, the complex system of contextual factors, and ultimately determines whether parent carers are empowered or disempowered.

A key component of the interaction between parent carers and service providers is the way that information is shared, managed and understood. The following section explores this in detail.

### 4.2 Information Management

In healthcare settings, it is a common belief that a well‐informed patient or carer is an empowered one [45]. This often carries into clinical interactions, where it is typically assumed to be the responsibility of the service provider to ‘provide’ that information [45]. While also reflected in the stories of the participants in our study, we argue that these assumptions are shaped by underlying standards and expectations tied to parent carer and service provider roles, which are constructed and enacted within the complex system.

**Figure 2:**
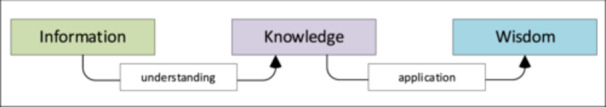
Information, knowledge and wisdom.

Our findings support that simply providing or granting access to information may not be sufficient to empower parent carers [45]. Drawing on concepts from information science, particularly the widely referenced DIKW (Data/Information/Knowledge/Wisdom) pyramid [46], we propose that parent carers need to process and make sense of this information, if they are to apply it in meaningful and practical ways (Figure 2).

Viewing information management through this lens may be particularly helpful in today's society, where parent carers have seemingly unmetered access to vast amounts of information from the internet [47]. We suggest that this may demand an evolution of the service provider role, from simply delivering information to managing misinformation and facilitating understanding. This transformation would require service providers to recognise that they can no longer be, nor perhaps should they be, gatekeepers of information. This may pave the way for more genuine bi‐directional sharing of information, and challenge service providers to be more thoughtful and open about how they process and apply the information that parent carers bring to the consultation. We argue that it is only when service providers allow information shared by parent carers to meaningfully influence their practice that space is created for true parental agency. Ultimately, this action is critical to empowering parent carers, perhaps more so that simply providing access to information or supporting the development of their knowledge and skills.

### 4.3 Implications for Practice and Future Work

We believe that the model we present offers a framework for service providers to actively reflect on their interactions and behaviours with parent carers, and to explore the various forces that have them feeling they should be behaving and acting in certain ways. This exploration may help to resolve feelings of role uncertainty and shaky professional identity, providing the space and confidence to move beyond the security of traditional forms of practice that locate power and control with the service provider. This should include giving greater attention to the impact of their interaction with parent carers, in particular how they engage with information that is shared by parent carers.

Currently, most initiatives to promote parent carer empowerment aim to target change in parent carer behaviours [20]. We suggest an alternative or complementary approach could be to develop initiatives for service providers, potentially using our model to support critical reflection of their role in parent carer empowerment.

We recommend that future research on parent carer empowerment would be supported by developing a contemporary consensus definition that reflects its complexity.

## 5 Strengths and Limitations

A strength of our study is it's methodological integrity, which we have reported comprehensively and with transparency. Furthermore, the study was planned and delivered in collaboration with parent carer research partners, who have been fully involved in data generation and data analyses. We believe this has contributed to a greater depth of insight and interpretation of the data.

One limitation of this study is that it was conducted within a single NHS organisation. Whilst we acknowledge this means our findings are context specific, feedback from our parent carer partners living in other areas suggests that they are likely to resonate more broadly.

Another limitation of this study is that we have not explored the implications of the different interviewer/interviewee dynamics that were created during data generation. We believe this is an interesting and important phenomena that warrants further exploration.

## 6 Conclusion

Our paper offers new insights into the complex ways contextual factors and systemic forces shape parent carer empowerment. By examining the construction and performance of roles within the interaction between parent carers and service providers, we highlight the critical importance of relational dynamics in fostering or constraining empowerment. Importantly, our findings challenge conventional assumptions about the role of information in this process. We argue that true empowerment occurs not simply when parent carers receive information or acquire knowledge, but when the information they provide is recognised, understood, and meaningfully applied by service providers. This reframing has important implications for practice, underscoring the need for more reciprocal, inclusive, and collaborative models of care that genuinely centre the voices and expertise of parent carers.

## Author Contributions

**Jim Reeder:** conceptualisation, methodology, writing – review and editing, funding acquisition, investigation, writing – original draft, formal analysis, project administration, data curation, software, resources. **Saira Minhas:** conceptualisation, investigation, writing – review and editing, formal analysis, data curation. **Sharon Foxwell:** conceptualisation, investigation, writing – review and editing, formal analysis, data curation. **Mark Williams:** conceptualisation, investigation, writing – review and editing, formal analysis, data curation. **Jane R. Smith:** conceptualisation, methodology, writing – review and editing; supervision. **Sally Kendall:** conceptualisation, methodology, writing – review and editing, supervision. **Christopher Morris:** conceptualisation, writing – review and editing, methodology, supervision.

## Ethics Statement

Cambridge South Research Ethics Committee approved the study and research materials on 08/02/24 (ref: 24/EE/0020).

## Conflicts of Interest

The authors declare no conflicts of interest.

## Supporting information

Supporting File 1

Supporting File 2

## Data Availability

The data that support the findings of this study are available on request from the corresponding author. The data are not publicly available due to privacy or ethical restrictions.
