# Supplementary material for: Exploring Parent Carer Empowerment in Childhood Onset Disability: A Qualitative Study
Source: Health Expect. 2026 Aug 5;29(4):e70808. doi: 10.1111/hex.70808 (PMC13440351; doi:10.1111/hex.70808)
Supplement: Supplementary file 1 [file HEX-29-e70808-s001.docx]

### Supplementary Material 2: Provisional Interview Schedule and Focus Group Plan

- A group of parent carers and researchers have developed a vision of what parent carer empowerment is.

“Parent carers feel listened to and have influence in a collaborative partnership with health professionals. This supports them to make decisions and take actions about their child’s care.”

What are your thoughts about this vision?

- What do you think are the barriers to realising this vision?
- How do you think we would know when parent carers are empowered?
- What do you think needs to happen for parent carers to become empowered?
