# Supplementary material for: Exploring Parent Carer Empowerment in Childhood Onset Disability: A Qualitative Study
Source: Health Expect. 2026 Aug 5;29(4):e70808. doi: 10.1111/hex.70808 (PMC13440351; doi:10.1111/hex.70808)
Supplement: Supplementary file 2 [file HEX-29-e70808-s002.docx]

### Supplementary Material 1: Eligibility Criteria

|  | Parent Participants | Other Stakeholder Participants |
| --- | --- | --- |
| Inclusion Criteria | Any parent carer of a child (aged 0-19 years) accessing children’s services from the hosting NHS trust. | Any health professional or support staff working directly with children and their families in the hosting NHS trust.  Or  Any person responsible for managing health services for children in the hosting NHS trust.  Or  Any person responsible for commissioning health services for children in the hosting NHS trust. |
| Exclusion Criteria | None | Newly qualified clinical staff (under 12 months clinical) experience. |
